# The Frontline Immunotherapy-Based Treatment of Advanced Clear Cell Renal Cell Carcinoma: Current Evidence and Clinical Perspective

**DOI:** 10.3390/biomedicines10020251

**Published:** 2022-01-24

**Authors:** In-Ho Kim, Hyo Jin Lee

**Affiliations:** 1 Department of Internal Medicine, Division of Medical Oncology, Seoul St. Mary’s Hospital, The Catholic University of Korea, College of Medicine, Seoul 06591, Korea; ihkmd@icloud.com; 2 Department of Internal Medicine, Chungnam National University School of Medicine, Daejeon 35015, Korea

**Keywords:** immunotherapy, renal cell carcinoma, systemic treatment

## Abstract

Approximately 400,000 patients are diagnosed with kidney cancer annually worldwide, leading to approximately 170,000 deaths. Renal cell carcinoma (RCC) accounts for more than 90% of kidney cancers. The most common histological subtype is clear cell RCC, which is found in approximately 85% of metastatic RCC cases. The VHL-HIF-VEGF axis is well known; therefore, targeting VEGF has been the mainstay for managing advanced clear cell RCC. Recently, the treatment landscape for advanced clear cell RCC has changed extensively. In particular, immune checkpoint inhibitor-based treatment showed promising results in front-line treatment and became the standard of care. Herein, we review the current evidence on front-line treatment options and discuss the clinical and future perspective.

## 1. Introduction

Kidney cancer is diagnosed in approximately 400,000 patients annually worldwide, leading to approximately 170,000 deaths [1]. Renal cell carcinoma (RCC) accounts for more than 90% of kidney cancers, and the most common histological subtype is clear cell RCC (ccRCC), which is found in approximately 85% of metastatic RCC cases [2]. ccRCC is closely associated with a loss-of-function mutation in the *von Hippel–Lindau (VHL)* gene [3]. Loss of the *VHL* gene leads to the upregulation of hypoxia inducible factor (HIF) and resultant overexpression of hypoxia-inducible genes, such as vascular endothelial growth factor (VEGF), platelet-derived growth factor-β (PDGF-β), and transforming growth factor-α (TGF-α) (VHL-HIF-VEGF axis), which are involved in tumorigenesis and the progression of ccRCC [4]. Therefore, targeting angiogenesis was hypothesized to be especially important in ccRCC, and several clinical trials have shown that targeted therapies against the VEGF signaling axis (sunitinib, pazopanib, axitinib, cabozantinib, etc.), have provided significant clinical benefits for patients with ccRCC [5,6,7,8,9]. However, the duration of the antitumor effects of these agents is not long, and resistance eventually develops during VEGF inhibitor (VEGFi) treatment [10].

**Figure 1:**
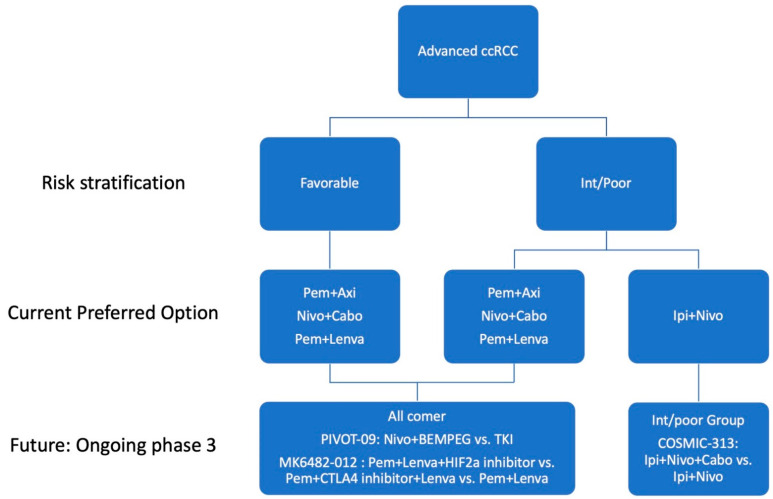
Current preferred treatment options and ongoing large-scale phase 3 trials in the first line setting of ccRCC. Axi, axitinib; BEMPEG, bempegaldesleukin; Cabo, cabozantinib; ccRCC, clear cell renal cell carcinoma; CTLA4, cytotoxic T-lymphocyte antigen 4; HIF2a, hypoxia inducible factor-2 alpha; Int, intermediate; Ipi, ipilimumab; Lenva, lenvatinib; Nivo, nivolumab; Pem, pembrolizumab; TKI, tyrosine kinase inhibitor.

RCC is well known as an immunogenic tumor [11]. Immunotherapy such as high-dose interleukin-2 (IL-2) and interferon-alpha (IFN-*α*) showed clinical activity, but it was very modest, and only a few patients achieved a dramatic and durable response [12,13,14]. Recently, immune checkpoint inhibitors (ICIs) and combination strategies have changed the landscape of treatment for patients with advanced ccRCC [15]. Herein, we discuss the current evidence of the front-line treatment landscape of metastatic ccRCC, its clinical considerations and future perspective (Figure 1).

## 2. Immune Checkpoint Inhibitor Combinations

CheckMate 214 was a phase 3 trial that compared ipilimumab (fully human anti-cytotoxic T-lymphocyte antigen 4 (CTLA-4) monoclonal antibody) plus nivolumab (fully human anti-programmed death 1 (PD-1) monoclonal antibody) (Ipi+Nivo) with sunitinib for previously untreated ccRCC [16]. The co-primary endpoints were overall survival (OS), objective response rate (ORR) and progression-free survival (PFS) in patients with International Metastatic Renal Cell Carcinoma Database Consortium (IMDC) intermediate or poor risk. At a median follow-up of 25.2 months in intermediate- and poor-risk patients, the 18-month OS rate was higher in the Ipi+Nivo than in the sunitinib group (75% vs. 60%). Ipi+Nivo achieved longer OS (not reached (NR) vs. 26 months) and superior ORR (42% vs. 27%) compared with sunitinib. The median PFS with Ipi+Nivo and sunitinib was 11.6 months and 8.4 months, respectively (not significant). Meanwhile, among IMDC favorable-risk patients, the ORR was lower with Ipi+Nivo than with sunitinib (29% vs. 52%), and the median PFS was also shorter with Ipi+Nivo than with sunitinib (15.3 vs. 25.1 months). In overall population, grade 3 or higher adverse events (AEs) occurred in 46% of the Ipi+Nivo and 63% of the sunitinib group. AEs leading to treatment discontinuation occurred in 22% of the Ipi+Nivo and 12% of the sunitinib group. The most common AEs of grade 3 or higher were increased lipase level (10%), diarrhea (4%), and fatigue (4%). In exploratory analyses, the benefits of OS and ORR were observed with Ipi+Nivo compared to sunitinib among intermediate- and poor-risk patients across tumor PD-L1 expression levels, although the magnitude of benefit was higher in patients that were PD-L1 positive (≥1%). Meanwhile, Ipi+Nivo achieved longer PFS than with sunitinib in PD-L1 positive, not in PD-L1 negative group. Recent reports with extended follow-up showed that the benefit of Ipi+Nivo was maintained to be superior to sunitinib in terms of ORR, PFS and OS in patients with intermediate/poor risk [17,18].

## 3. Immune Checkpoint Inhibitor with VEGF Inhibitors

Combination with other therapies, including anti-angiogenic agents, is an important strategy currently under investigation to enhance the clinical benefits of ICIs. Anti-angiogenic treatment may promote T-cell priming and activation via dendritic cell maturation [19,20], normalize the tumor vasculature for increased T-cell tumor infiltration [21,22], and establish an immunoreactive tumor microenvironment by decreasing myeloid-derived suppressor cells (MDSCs) and Treg populations [23,24]. Therefore, immunotherapy with T-cell activation may be enhanced through the reversal of VEGF-mediated immunosuppression.

KEYNOTE-426 was an open-label, phase 3 trial that compared pembrolizumab (humanized anti-PD-1 monoclonal antibody) plus axitinib (Pem+Axi) with sunitinib for previously untreated advanced ccRCC [25]. OS and PFS were primary endpoints. After a median follow-up of 12.8 months, the 12-month survival rate was higher in the Pem+Axi group than in the sunitinib group (89.9% vs. 78.3%). The Pem+Axi group showed significantly extended PFS compared with the sunitinib group (15.1 vs. 11.1 months). The ORR was higher in the Pem+Axi than in the sunitinib (59.3% vs. 35.7%) group. The benefit of Pem+Axi with respect to OS and PFS was observed in all subgroups, including patients with IMDC risk and PD-L1 expression. Grade 3 or higher AEs occurred in 75.8% of patients in the Pem+Axi group and 70.6% in the sunitinib group. AEs of grade 3 or higher that occurred in 10% or more of the patients were hypertension (22%) and increased alanine aminotransferase levels (13%) in the Pem+Axi group. The extended follow-up analyses demonstrated the continued clinical benefit of Pem+Axi in terms of OS and PFS [26,27].

The phase 3 trial, JAVELIN Renal 101, compared avelumab (fully human anti-PD-L1 monoclonal antibody) plus axitinib (Avel+Axi) with sunitinib in previously untreated advanced ccRCC [28]. OS and PFS among tumor PD-L1 positive patients were co-primary endpoints. The Avel+Axi group achieved significantly longer PFS than the sunitinib group among patients with PD-L1-positive tumors (13.8 vs. 7.2 months) and the overall population (13.8 vs. 8.4 months). Among the patients with PD-L1–positive tumors, there was no difference in OS between the two groups. The Avel+Axi group showed higher ORR than the sunitinib (55.2% vs. 25.5%) group among patients with PD-L1–positive tumors. Grade 3 or higher AEs occurred in 71% of the patients in both groups. The most common AEs of grade 3 or higher were hypertension (26%), diarrhea (7%), increased alanine aminotransferase levels (6%), and palmar–plantar erythrodysesthesia syndrome (6%). Updated efficacy results still demonstrated that Avel+Axi showed a significant PFS and ORR benefit compared with sunitinib [29]. This benefit was observed across several subgroups, including all patients with IMDC risk. However, OS was still not different between the two groups.

IMmotion151 was a multicenter, open-label, phase 3, randomized controlled trial that compared atezolizumab (fully human anti-PD-L1 monoclonal antibody) plus bevacizumab (Atezo+Bev) with sunitinib in previously untreated metastatic RCC with a component of clear cell or sarcomatoid histology [30]. Co-primary endpoints were PFS in the PD-L1-positive and OS in the intention-to-treat (ITT) patients. In the PD-L1-positive patients, Atezo+Bev achieved longer PFS than sunitinib (11.2 vs. 7.7 months). The PFS benefit of Atezo+Bev was observed in patients across key clinical subgroups, including Memorial Sloan-Kettering Cancer Center (MSKCC) risk groups, sarcomatoid histology, liver metastasis, and previous nephrectomy. Notably, the depth of PFS benefit was very impressive in sarcomatoid histology (HR, 0.46 in the PD-L1-positive and 0.56 in the ITT patients). However, there was no significant difference in OS (33.6 vs. 34.9 months) in the ITT population. Atezo+Bev exhibited a favorable safety profile. Atezo+Bev was associated with fewer severe treatment-related AEs and a lower regimen discontinuation rate (5% vs. 8%) than sunitinib. In the final analysis with extended follow-up, there was no OS difference between Atezo+Bev and sunitinib [31].

CheckMate 9ER was a randomized phase 3, open-label trial that compared nivolumab plus cabozantinib (Nivo+Cabo) with sunitinib alone in previously untreated advanced ccRCC [32]. PFS was the primary endpoint. Nivo+Cabo had a superior PFS over sunitinib (16.6 vs. 8.3 months). Nivo+Cabo showed a significant OS benefit compared to sunitinib. The 1-year OS rate was higher in the Nivo+Cabo group than in the sunitinib group (85.7% vs. 75.6%). ORR was higher with Nivo+Cabo than with sunitinib (55.7% vs. 27.1%); a CR was observed in 8.0% and 4.6% of patients in the Nivo+Cabo and sunitinib groups, respectively. The benefits of Nivo+Cabo over sunitinib with respect to PFS and OS were observed across several subgroups, including patients with IMDC risk, tumor PD-L1 positive status, and presence of bone metastases. Grade 3 or higher AEs occurred in 75.3% and 70.6% of the patients in the Nivo+Cabo and sunitinib groups, respectively. The most common AEs of grade 3 or higher were hypertension (13%), hyponatremia (9%), palmar–plantar erythrodysesthesia syndrome (8%), diarrhea (7%), and increased lipase level (6%). Patients in the Niv+Cabo group reported statistically better health-related quality of life (QoL) than those in the sunitinib group [33]. 

CLEAR was a randomized phase 3, open-label trial that compared pembrolizumab plus lenvatinib (Pem+Lenva), lenvatinib plus everolimus (Lenva+Ever), or sunitinib alone in previously untreated advanced ccRCC [34]. The primary endpoint was the PFS. PFS was significantly longer in the Pem+Lenva group than in the sunitinib group (23.9 vs. 9.2 months) and was significantly longer in the Lenva+Ever group than in the sunitinib group (14.7 vs. 9.2 months). OS was longer with Pem+Lenva than with sunitinib but was not longer with Lenva+Ever compared with sunitinib. The benefit of Pem+Lenva versus sunitinib with respect to PFS and OS was generally observed across several subgroups, including patients with IMDC risk, PD-L1 expression, and sarcomatoid features. The ORR was 71.0% with Pem+Lenva, 53.5% with Lenva+Ever, and 36.1% with sunitinib. The CR rate was 16.1%, 9.8%, and 4.2%, respectively, in the Pem+Lenva, Lenva+Ever, and sunitinib groups. Grade 3 or higher AEs occurred in 82.4%, 83.1%, and 71.8% of patients in the Pem+Lenva, Lenva+Ever, and sunitinib groups, respectively. In the Pem+Lenva group, the most common grade 3 or higher AEs were hypertension (28%), diarrhea (10%), weight decrease (8%), and proteinuria (8%), and AEs led to discontinuation of any drug in 37.2% of the patients (Lenva, 25.6%; Pem, 28.7%; both drugs, 13.4%), led to dose reduction of Lenva in 68.8% of the patients, and led to the interruption of any drug in 78.4% of the patients. A recent study on QoL demonstrated that Pem+Lenva showed similar or improved health-related QoL and disease-related symptom scores compared with sunitinib [35]. 

To date, the NCCN and EAU guidelines [36,37] recommend Pem+Axi, Nivo+Cabo, and Pem+Lenva as preferred first-line treatments in both favorable and intermediate/poor groups. Ipi+Nivo is recommended as a preferred first-line treatment in the intermediate/poor group. We summarized the results of frontline ICI-based trials in Table 1.

## 4. Biomarkers for ICI-Based Treatment in RCC

### 4.1. PD-L1 Expression

Several previous studies have suggested that high PD-L1 expression and tumor-infiltrating lymphocytes may be associated with poor prognosis in RCC [39,40]. Increased tumor cell PD-L1 or PD-L1 plus tumor CD8-positive T-cell counts were associated with poor prognosis in patients with metastatic RCC receiving VEGFi [41,42]. Therefore, PD-L1 expression has been extensively studied as a predictive biomarker for ICI-based treatment in advanced ccRCC. 

In CheckMate 214 [16], additional exploratory endpoints included outcomes according to the level of tumor PD-L1 expression (≥1% vs. <1%), as assessed at a central laboratory using the Dako PD-L1 IHC 28-8 pharmDx test. Ipi+Nivo showed PFS benefit compared with sunitinib among patients with PD-L1 positive tumors (PFS, 22.8 vs. 5.9 months) but not among those with PD-L1 negative tumors (11.0 vs. 10.4 months). In contrast, Ipi+Nivo showed longer OS and higher ORR than sunitinib among intermediate or poor-risk patients across tumor PD-L1 expression levels, although the depth of benefit was deeper in the patients with PD-L1 positive tumors. This result suggests that the clinical benefit of Ipi+Nivo is prominent in PD-L1 positive tumors, but PD-L1 expression is not a predictive biomarker because the clinical benefit of Ipi+Nivo is also observed in PD-L1 negative patients. 

PD-L1 expression has also been extensively investigated in ICI with VEGFi. In KEYNOTE-426, PD-L1 expression was assessed using the PD-L1 IHC 22C3 pharmDx assay (Agilent Technologies) and was characterized according to the combined positive score (CPS) [25]. A total of 60.5% of patients had a CPS of 1 or higher. In PD-L1 positive (CPS 1 or higher) patients, Pem+Axi showed higher 1-year OS rates than sunitinib (90.1% vs. 78.4%). This benefit of Pem+Axi in terms of 1-year OS rates in the PD-L1 negative group was also consistent (91.5% vs. 78.3%). Median PFS was longer with Pem+Axi than with sunitinib in PD-L1 positive groups (15.3 vs. 8.9 months) and in PD-L1 negative groups (15.0 vs. 12.5 months). Therefore, the benefits of Pem+Axi with respect to OS and PFS were consistent across the PD-L1 expression. 

In IMmotion151, PD-L1 expression was evaluated using the VENTANA PD-L1 SP142 assay [30]. Tumor-infiltrating immune cells expressing PD-L1 (1% or higher) were defined as positive. Atezo+Bev showed PFS benefit in the PD-L1 positive group, but not in the PD-L1 negative group. The PFS of the Atezo+Bev group showed a gradient of increasing benefit with increasing PD-L1 expression. 

In CheckMate 9ER, PD-L1 expression was defined as the percentage of positive tumor cells based on the Dako PD-L1 IHC 28-8 pharmDx assay [32]. PD-L1 positive tumors were defined as tumor cells ≥ 1%. Clinical benefit of Nivo+Cabo with respect to PFS, OS and ORR was consistent across PD-L1 status. Interestingly, the median PFS of Nivo+Cabo was longer in the PD-L1 negative than in the positive group (17.7 vs. 11.9 months). 

In the CLEAR study, PD-L1 expression was assessed using the PD-L1 IHC 22C3 pharmDx assay (Agilent Technologies) and reported as the CPS [34]. PD-L1 positivity was defined as CPS 1 or higher, and approximately 30% of patients were positive. Clinical benefit of Pem+Lenva with respect to PFS and OS was consistent across PD-L1 status.

Given the results of the exploratory analyses of pivotal trials (Table 2), PD-L1 expression showed inconsistent predictive effects across trials. Furthermore, PD-L1 testing methods and cutoffs were different across trials; therefore, further studies are needed. To date, PD-L1 expression cannot be used as a definitive biomarker in ICI-based front-line treatment.

### 4.2. Sarcomatoid Differentiation

Sarcomatoid differentiation is known to occur in 10% of RCC and is associated with more aggressive features, poor response to VEGFi, and higher PD-1/PD-L1 expression [43]. A previous study showed that angiogenesis gene expression is low, and PD-L1 expression and T-effector gene signatures are high in sarcomatoid tumors [44,45].

A post hoc analysis of CheckMate 214 evaluated outcomes in patients with sarcomatoid features (sRCC) [46]. A total of 139 patients with intermediate/poor-risk disease and 6 with favorable-risk disease were identified as having sRCC. The Ipi+Nivo group showed significantly longer OS (NR vs. 14.2 months) and PFS (26.5 vs. 5.1 months) than the sunitinib group. The ORR was significantly higher in the Ipi+Nivo than in the sunitinib group (60.8% vs. 23.1%), with CR rates of 18.9% and 3.1%, respectively. 

A subgroup analysis of KEYNOTE-426 evaluated outcomes in patients with sRCC [47]. Sarcomatoid features were present in 18.2% of the patients. Pem+Axi improved OS (12-month survival rate, 83.4% vs. 79.5%), PFS (NR vs. 8.4 months), ORR (58.8% vs. 31.5%), and CR rates (11.8% vs. 0%) over sunitinib in patients with sRCC. 

The updated analysis of CheckMate 9ER evaluated outcomes by sarcomatoid histology [48]. Nivo+Cabo improved PFS (10.9 vs. 4.2 months), OS (NR vs. 19.7 months) and ORR (55.9% vs. 22.0%) over sunitinib in sRCC. Notably, in terms of HR, prominent benefits of Nivo+Cabo with respect to PFS (HR, 0.39 vs. 0.54) and OS (HR, 0.36 vs. 0.68) were observed in patients with sRCC compared to those without sRCC.

A subgroup analysis of CLEAR evaluated the outcomes by sarcomatoid feature [49]. PFS results favored Pem+Lenva over sunitinib (11.1 vs. 5.5 months) treatment in patients with sRCC.

A recent meta-analysis including four RCTs (CheckMate 214, KEYNOTE-426, IMmotion151, and JAVELIN Renal 101) evaluated the outcomes of ICI-based treatment in sRCC [50]. ICI-based treatments were associated with an improved PFS, OS, and ORR compared with sunitinib, with a reduction of more than 40% in progression (HR, 0.56) and mortality (HR, 0.56) risk. Finally, ICI-based treatment significantly increased the possibility of obtaining CR (relative risk, 8.15; *p* = 0.0002) with an incidence of 11%. 

The ORR and CR rates of the ICI-based regimen are summarized in Table 3. Given these results, ICI-based treatment should be considered as a front-line treatment in patients with sarcomatoid differentiation.

### 4.3. Tumor Mutation Burden (TMB)

TMB has been extensively studied as a predictive biomarker for ICI. Prospective biomarker analysis of the KEYNOTE-158 study suggested that high TMB (TMB-H) could be a predictive biomarker for pembrolizumab in advanced solid tumors [52]. On 6 June 2020, the FDA granted accelerated approval of pembrolizumab to treat adult and pediatric patients with unresectable or metastatic TMB-H (≥10 mutations/megabase) solid tumors that have progressed following prior treatment and who have no satisfactory alternative treatment options. Meanwhile, RCC, which is recognized as an immunogenic tumor, has a relatively lower TMB than other immunogenic tumors, including melanoma, lung cancer, and bladder cancer [53]. A previous study analyzed the clinical and genomic data of 1662 advanced cancer patients treated with ICIs using targeted next-generation sequencing (MSK-IMPACT) [54]. The study suggested that higher somatic TMB is associated with better OS after ICI treatment for most cancers, but this association of higher TMB and improved survival was not observed in patients with RCC. Several other retrospective studies also did not find an association between TMB and prognosis in RCC patients who were treated with ICIs [55,56]. In addition to TMB with an NGS-based technique, a previous study investigated whether the frameshift nature of insertion/deletion (indel) mutations creates tumor-specific neoantigens and contributes to the immunogenic phenotype in the pan-cancer dataset [57]. Whole-exome sequencing data from 5777 solid tumors were analyzed. Among the pan-cancer cohort, the highest proportion and number of indel mutations was observed in RCC. Compared with single nucleotide variation (SNV) mutations, it was observed that indel mutations generate 3 times more high-binding affinity neoantigens and 9 times more mutant-specific binders. This study also showed that indel load was significantly associated with ICI response compared with non-SNV load in melanoma cohorts. These approaches were applied in several pivotal clinical trials. The exploratory analysis of IMmotion150, a randomized phase 2 study of atezolizumab alone or combined with bevacizumab versus sunitinib, showed that TMB, tumor neoantigen burden (TNB), and indels were not associated with PFS in any of the three treatment arms [58]. Recently, biomarker analysis of CheckMate 214 also reported that TMB and tumor indel burden were not associated with survival benefit in patients treated with Ipi+Nivo [59]. A biomarker study of JAVELIN Renal 101 also did not show an association between PFS and TMB in both the Avel+Axi and sunitinib arms [60]. Given these results, TMB, TNB, and indel burden need more study as biomarkers for ICI-based treatment in RCC.

### 4.4. RNA Gene Signature

The IMmotion150 biomarker study performed whole transcriptome profiling of 263 patients using RNA sequencing and showed distinct biological subgroups based on relative expression levels of angiogenesis (Angio), immune (T-effector presence and function, IFN-γ response, checkpoint inhibitors, and antigen presentation), and myeloid inflammation-associated genes [58]. The angio-high signature group was characterized by relatively higher vascular density, whereas high expression of T-effector gene signature was positively associated with PD-L1 expression and CD8 T-cell infiltration. In the sunitinib arm, ORR was significantly higher in the angio-high than -low group (46% vs. 9%). Increased PFS was observed in the angio-high group than in the angio-low group. In contrast, in the Atezo+Bev arm, the ORR was higher in the T-effector-high group than in the -low group (49% vs. 16%). Additionally, Atezo+Bev showed longer PFS in the T-effector-high group than the -low group. Meanwhile, high myeloid inflammation gene signature expression (Myeloid-High), which is associated with suppression of the antitumor adaptive T-cell response, was associated with reduced PFS in the atezolizumab monotherapy group (HR 2.98) and, to a lesser extent, in the Atezo+Bev group (HR, 1.71), but not in the sunitinib group. Based on this result, the authors suggested that the addition of VEGFi may overcome innate inflammation-mediated resistance. An exploratory study of IMmotion151 reported similar results [44]. T-effector-high was associated with improved PFS in the Atezo+Bev group compared with the sunitinib group. Angio-high was associated with improved PFS in the sunitinib arm. Atezo+Bev improved PFS compared to sunitinib in the angio-low group. Interestingly, the angio signature was higher in the favorable than in the intermediate/poor MSKCC risk groups, which could explain the prominent benefit of ICI in the intermediate/poor risk group compared with the favorable group. The biomarker analysis of JAVELIN Renal 101 generated the whole-transcriptome profiles of 720 patients and defined 26 immune gene signatures (Renal 101 immuno [R-101Immuno]) and 26 gene angio-signatures (Renal 101 angio [R-101Angio]) [60]. Patients with R-101Immuno-high showed longer PFS than those with R-101Immuno-low signatures in the Avel+Axi arm, but the signature did not differentiate PFS in the sunitinib arm. The R-101Angio-high group showed improved PFS in the sunitinib arm, but not in the Avel+Axi arm. CheckMate 214 performed survival analysis using six different gene expression signatures [59]. An angio-high signature (per IMmotion 150 signature) was associated with improved ORR and PFS benefits for sunitinib, and an angio-low signature was associated with a higher ORR in the Ipi+Nivo group. However, immune signature scores were not predictive of ORR in both the Ipi+Nivo and sunitinib groups. 

Given these results, although the results of the above-mentioned studies are not consistent, RNA gene signature can be a potential biomarker if further studies with detailed validation are conducted. 

In addition to the above, there have been many studies on biomarkers of RCC, including *PBRM1* mutation [29,59,61,62,63], endogenous retroviruses [64,65,66,67], and the neutrophil–lymphocyte ratio [68,69]. Although there are no definitive biomarkers of clinical impact, many studies are currently ongoing. We hope that these studies will improve the prognosis and treatment outcomes of advanced ccRCC.

## 5. How to Select the First-Line Treatment

### 5.1. Favorable-Risk Group

In the favorable-risk group, several international guidelines [36,37] recommend Pem+Axi, Nivo+Cabo, or Pem+Lenva, but not Ipi+Nivo, as the preferred frontline treatment options in advanced ccRCC. CheckMate 214 demonstrated the clinical benefit of Ipi+Nivo in the IMDC intermediate/poor risk group. In contrast, the ORR (29% vs. 52%) and PFS (median, 15.3 vs. 25.1 months) were improved with sunitinib over Ipi+Nivo in the favorable-risk group [16]. This superiority of sunitinib over Ipi+Nivo with respect to ORR and PFS in the favorable-risk group was maintained in the extended follow-up results [17,18]. OS was not significantly different, but 30-month OS rates were slightly higher in the sunitinib group (85% vs. 80%). Therefore, the Ipi+Nivo combination is not recommended as the preferred option in the favorable-risk group. In contrast to the ICI combination, ICI with an VEGFi demonstrated clinical benefit in the favorable-risk group. In subgroup analyses of previous pivotal trials, PFS and ORR benefits with ICI+VEGFi were observed to be more favorable than sunitinib [26,30,34,47,58]. However, these trials did not show the OS benefit of ICI+VEGFi over sunitinib alone. It is not clear whether ICI+VEGFi treatment should be performed in all favorable-risk patients while enduring the toxicity and cost of ICI+VEGFi, because some patients with favorable risk achieved durable disease control with pazopanib or sunitinib alone, or even without systemic treatment [70,71]. Although selection criteria for VEGFi alone or ICI+VEGFi have not been validated, VEGFi alone may be another treatment option in selected patients, such as those with slowly progressing, asymptomatic, and low-volume disease. However, ICI+VEGFi should be considered for patients with a high tumor burden, clinically rapid progression, or symptomatic disease requiring immediate disease control. Moreover, ICI+VEGFi provided a better ORR than sunitinib in several pivotal trials, and we should check the OS results with long-term follow-up in the future to develop guidelines for drug choice in the favorable-risk group.

### 5.2. Intermediate/Poor-Risk Group

In the intermediate/poor group, several international guidelines [36,37] recommend Ipi+Nivo, Pem+Axi, Nivo+Cabo, or Pem+Lenva as preferred frontline treatment options in advanced ccRCC. The choice of treatment options is either Ipi+Nivo or ICI+VEGFi. There has been no head-to-head trial between Ipi+Nivo and ICI+VEGFi treatments. Although it is impossible to compare these trials directly, several issues must be considered for decision-making in practice. In patients with symptomatic, high disease burden who require rapid disease control, ICI+VEGFi can be a better option than Ipi+Nivo. ICI+VEGFi achieves numerically higher ORR and shorter time to response, which can control disease rapidly. The low PD rate is also an important advantage of ICI+VEGFi. Meanwhile, Ipi+Nivo can be an important option for patients who do not need rapid tumor reduction with clinicians who can manage immune-related AEs well. Ipi+Nivo demonstrated a durable response and long-term survival, which has not been clearly demonstrated in ICI+VEGFi trials due to the shorter follow-up duration of these trials. Therefore, we should check the updated results for survival with extended follow-up of ICI+VEGFi trials. The toxicity profiles were different between Ipi+Nivo and ICI+VEGFi. Ipi+Nivo can induce higher rates of immune-related AEs, which can be fatal and require high doses of steroids. Meanwhile, ICI+VEGFi can cause not only ICI-induced toxicities but also VEGFi-induced chronic toxicities resulting in a decreased QoL.

The next important issue is the choice across several ICI+VEGFi treatments. There are no head-to-head studies, and there is no definite consensus on the choice of treatment. Numerically, Pem+Lenva provides impressive ORR with a high CR rate and PFS; therefore, Pem+Lenva could be considered in patients who need rapid tumor reduction or have a strong desire for CR, regardless of toxicity. Meanwhile, Nivo+Cabo and Pem+Axi are good options for patients who need tolerable treatment with good response. Pem+Axi and Nivo+Cabo showed numerically lower rates of AEs, which led to drug discontinuation compared to Pem+Lenva (7% and 6% vs. 13%). In particular, Nivo+Cabo showed improved quality of life compared to sunitinib [33], while Pem+Lenva showed a similar QoL to sunitinib [35]. Avel+Axi and Atezo+Bev are less preferred options because these regimens have not shown OS benefits. Toxicity profiles differ across ICI+ VEGFi. Pem+Lenva appeared to cause hypertension, proteinuria, and increased lipase. Meanwhile, palmar–plantar erythrodysesthesia syndrome was more prominent in the Nivo+Cabo group. Pem+Axi appeared to cause hepatotoxicity. 

Therefore, there are several considerations in determining treatment, so clinicians should consider these factors and choose the appropriate treatment.

## 6. Potential Candidates for Systemic Treatment and Ongoing Phase 3 Trials in Front-Line Setting

Several studies are investigating potential promising agents in RCC. In addition to PD-1/PD-L1 and CTLA4 inhibitors, several other immune checkpoints are also being investigated in many studies. For example, human endogenous retrovirus-H long terminal repeat-associating 2 (HHLA2) may be a promising checkpoint for the improvement of immunotherapy [72]. HHLA2, a member of the B7 family of immunoregulatory ligands, is known to have inhibitory effects on T cells and be overexpressed in RCC, and is associated with poor prognosis [73]. Recent study proposed that killer cell immunoglobulin-like receptor, three immunoglobulin domains and long cytoplasmic tail 3 (KIR3DL3) is an inhibitory receptor for HHLA2 in T cells and natural killer cells [74]. Moreover, this study showed that HHLA2 expression was not overlapping with PD-L1 expression in ccRCC, suggesting that HHLA2 may represent a regulated mechanism of tumor immune evasion that is independent of PD-L1. In addition, several other checkpoints such as LAG-3, TIM-3, and TIGIT [75] are currently being investigated in many other studies. 

Metabolic reprogramming is an important hallmark of tumor initiation and progression [76]. Constitutive HIF signaling results in extensive metabolic reprogramming including a shift towards increased glutamine utilization; therefore, RCC may be particularly susceptible to interference with glutamine metabolism [77,78]. Previous studies showed that glutamine utilization is increased in RCC and that tumor cells become addicted to glutamine [79,80]. Telaglenastat is a first-in-clinic, selective, potent glutaminase inhibitor, and previous early phase studies showed a tolerable safety profile and encouraging efficacy [81,82]. The CANTANA study, which was a recent phase 2 study of cabozantinib + telaglenastat with cabozantinib + placebo in previously treated patients, did not show clinical benefit from the addition of telaglenastat [83]. Currently, several studies are ongoing in other solid tumors as well as RCC, and future studies are warranted to determine the impact of glutaminase inhibition. 

Histone deacetylases (HDACs) are key regulators of gene expression that act as transcriptional repressors by removing acetyl groups from histones [84]. HDACs are grouped into four classes. Class I HDACs are frequently overexpressed in RCC, and RCC has lower protein acetylation levels than normal renal tissue [85]. Therefore, pan- or selective HDAC inhibition may be potential treatment strategy in RCC. Previous phase 1/2 trials of vorinostat, which is a class II HDAC inhibitor, reported that the combination of vorinostat with bevacizumab showed an ORR of 18% and manageable toxicity [86]. Another phase 1b study of abexinostat, which is a potent pan-HDAC inhibitor, showed an acceptable toxicity profile and encouraging anti-tumor activity [87]. In this study, markedly durable responses with abexinostat in combination with pazopanib were achievable in some patients that were refractory to previous VEGFi [88]. Currently ongoing is a phase 3 RENAVIV trial, which is investigating the efficacy and safety of pazopanib with abexinostat or placebo in the first-line setting [89].

Cyclin-dependent kinases (CDKs) are the families of protein kinases and play important roles in the control of cell division and modulation of transcription [90]. CDK4/6 play a role in phosphorylation of the retinoblastoma protein, which has been shown to be expressed in 95% of RCC [91]. In a xenograft model, abemaciclib, which is a potent CDK4/6 inhibitor, in combination with sunitinib reduced the size of the tumor [92]. Currently, a phase 1 study to evaluate the safety of the combination of abemaciclib and sunitinib is ongoing. In addition to the above-mentioned potential agents, many other studies of novel agents are ongoing. We summarized selected potential novel treatment trials in Table 4.

Additionally, in the first line setting, there have been several large-scale phase 3 trials of novel promising agents with ICI combinations including HIF-2a inhibitor [93], pegylated IL-2 (bempegaldesleukin) [94], and powerful triplet combinations [95]. We describe these strategies briefly.

### 6.1. HIF-2α Inhibitors

After the discovery of the role of the VHL-HIF-VEGF axis in ccRCC carcinogenesis, VEGF and mTOR inhibitors played an important role in ccRCC treatment; however, these agents showed drug resistance and disease progression. Recently, HIF has been recognized as a potential therapeutic target. HIF-1 and HIF-2 are heterodimeric transcription factors mediating the cellular response to hypoxia [96]. Although HIF-1α and -2α share some similar structures, it is known that their role in ccRCC may be different. ccRCC expressing only HIF-2α showed enhanced proliferation and resistance to replication stress compared with ccRCC expressing both HIF-2α and HIF-1α [97]. In xenograft models, downregulation of HIF-1α in HIF-1α proficient cell lines promotes tumor growth [98]; in contrast, downregulation of HIF-2α suppresses tumor formation [99]. These studies suggested that HIF-2α plays a major pro-tumorigenic role in human ccRCCs, whereas HIF-1α appears to function rather to inhibit aggressive tumor behavior [100]. From these backgrounds, inhibition of HIF-2α has been investigated actively. A phase 1 dose-escalation study of PT2385, a first-in-class HIF-2α antagonist, was conducted in heavily treated ccRCC patients [101]. This study showed a favorable safety profile and promising efficacy (ORR, 14%; disease control rates, 66%) in heavily treated patients. However, PT2385 was restricted by variable and dose-limited pharmacokinetics resulting from extensive metabolism of PT2385 to its glucuronide metabolite [102]. Belzutifan (PT2977, MK-6482), a 2nd generation small molecule HIF-2α inhibitor that showed increased potency and an improved pharmacokinetic profile, was evaluated in a phase 1 trial [103]. Recently, a phase 2 study evaluated the efficacy and safety of belzutifan in pre-treated patients with RCC with VHL disease [104]. Belzutifan showed promising results with a 49% ORR. In addition to tumor response, belzutifan showed promising response in non-RCC neoplasms. In sporadic ccRCC, a recent phase 1/2 study evaluated the efficacy and safety of belzutifan in pre-treated patients [105]. ORR was 25% and median PFS was 14.5 months. Among grade 3 AEs, anemia (27%) and hypoxia (16%) were the most common. Currently, several studies of belzutifan with/without other agents are ongoing in ccRCC patients [106,107,108]. In particular, in the first-line setting, phase 3 study to evaluate the efficacy and safety of pembrolizumab+belzutifan+lenvatinib or a co-formulation of pembrolizumab and quavonlimab (CTLA4 inhibitor)+lenvatinib versus pembrolizumab+lenvatinib is ongoing in advanced ccRCC [93]. 

### 6.2. Pegylated Interleukin-2

High-dose interleukin-2 (IL-2) shows durable response in RCC, but modest efficacy, treatment-associated toxicity and expansion of suppressive regulatory T cells (Tregs) limit its use in patients with RCC [109,110]. Bempegaldesleukin (BEMPEG) is a PEGylated IL-2 acting as a CD122-preferential IL-2 pathway agonist designed to activate and proliferate CD8+ T cells and NK cells [111,112]. In a phase 1 study, BEMPEG monotherapy was well-tolerated and showed clinical activity with objective response and durable disease control in heavily pretreated patients with advanced solid tumors [113]. Additionally, in a previous phase 1 study, BEMPEG+nivolumab showed promising ORR (71%) and manageable toxicity in untreated RCC patients [114]. Currently, a phase 3 PIVOT-09 trial to evaluate the efficacy and safety of BEMPEG+nivolumab vs. the investigator’s choice of TKI (sunitinib or cabozantinib) in patients with previously untreated advanced ccRCC is ongoing [94].

### 6.3. Powerful Combinations 

Dual combination strategies have shown promising results and become the standard of care in the first-line setting. Under the hypothesis that triple combinations may be more effective than dual combinations, triplet combination trials are ongoing now. COSMIC-313 is a phase-3 trial to compare Ipi+Nivo+cabozantinib with Ipi+Nivo+placebo in patients with previously untreated advanced ccRCC of intermediate or poor risk [95]. Another phase-3 study, to compare a co-formulation of pembrolizumab and quavonlimab (CTLA4 inhibitor)+Lenvatinib or pembrolizumab+belzutifan+lenvatinib vs. pembrolizumab+lenvatinib alone in the first-line setting, is also currently under way [93]. 

## 7. Conclusions

The landscape of frontline treatment for advanced ccRCC has changed in recent years. ICI-based treatment showed promising results, especially in the intermediate/poor risk group, and became the standard of care. Many studies are under way to improve the outcome of the current standard of care. The results of several ongoing trials for new treatments should be discussed in the near future. We believe that these efforts can improve patient prognosis.

## Figures and Tables

**Table 1:** Summary of pivotal trials of immune checkpoint inhibitor-based front-line treatments in advanced RCC.

	CheckMate 214 [16,18]		IMmotion151 [30,31]		KEYNOTE-426 [25,26]		JAVELIN Renal 101 [28,29,38]		CheckMate 9ER [32]		CLEAR [34]		
	Ipi+Nivo (*n* = 550)	Sun (*n* = 546)	Atezo+Bev (*n* = 454)	Sun (*n* = 461)	Pem+Axi (*n* = 432)	Sun (*n* = 429)	Avel+Axi (*n* = 442)	Sun (*n* = 444)	Nivo+Cabo (*n* = 323)	Sun (*n* = 328)	Pem+Lenva (*n* = 355)	Lenva+Ever (*n* = 357)	Sun (*n* = 357)
Primary endpoint	OS, PFS, ORR in int/poor		PFS in PD-L1 (+), OS in ITT		OS, PFS		OS, PFS in PD-L1 (+)		PFS		PFS		
IMDC risk group (%, favorable/intermediate/poor)	23/61/17	23/61/61	20/69/12 ^#^	20/69/12 ^#^	32/55/13	31/57/12	21/64/12	23/66/10	23/58/19	22/57/21	31/59/9	32/55/12	35/54/10
PD-L1 positive (%) ^&^	23%	25%	39%	40%	59%	62%	61%	65%	26%	25%	30%	33%	33%
Sarcomatoid feature (%)	14%	12%	15%	16%	18%	18%	NA	NA	11%	13%	8%	7%	6%
Median follow-up (months)	67.7		40		30.6		19.3		18.1		26.6		
Median OS (months)	55.7 *	38.4 *	36.1 *	35.3 *	NR	35.7	NR *	NR *	1yr OS rate: 86%	1yr OS rate: 76%	2yr OS rate: 79%	2yr OS rate: 66%	2yr OS rate: 71%
HR (95% CI)	0.72 (0.62–0.85)		0.91 (0.76–1.08)		0.68 (0.55–1.85)		0.80 (0.62–1.03)		0.60 (0.40–0.89)		0.66 (0.49–0.88)	1.15 (0.88–1.50)	1
Median PFS (months)	12.3 *	12.3 *	11.2 *	8.4 *	15.4	11.1	13.3 *	8.0 *	16.6	8.3	23.9	14.7	9.2
HR (95% CI)	0.86 (0.73–1.01)		0.83 (0.70–0.97)		0.71 (0.60–0.84)		0.69 (0.58–0.83)		0.51 (0.41–0.64)		0.39 (0.32–0.49)	0.65 (0.53–0.80)	1
ORR (%)	41	34	37	33	60	40	53	27	56	27	71	54	36
CR (%)	11	2	5	2	9	3	4	2	8	5	16	10	4
PR (%)	31	32	31	31	51	37	49	25	48	23	55	44	32
SD (%)	30	41	39	39	23	35	28	44	32	42	19	34	38
PD (%)	22	16	18	19	11	17	12	19	6	14	5	7	14
Grade 3 or higher TRAE (%)	47	64	40	54	67	62	71	72	75	71	82	83	72
Dose reduction (%)	NA	NA	NA	NA	20	30	42	43	56	52	69	73	50
Discontinuance rates (ICI/anti-VEGF/all) ^$^ (%)	22	12	2/5/5	8	21/20/7	12	NA/NA/8	13	7/8/6	17	29/26/13	22/25/19	14

*, the value of ITT, not primary endpoint population; ^#^, MSKCC prognostic group; ^$^, only to ICI+VEGFi trial; ^&^, PD-L1 testing method and cut-off are different across trials. Atezo, atezolizumab; Avel, avelumab; Axi, axitinib; Bev, bevacizumab; Cabo, cabozantinib; CI, confidence interval; CR, complete response; Ever, everolimus; HR, hazard ratio; ICI, immune checkpoint inhibitor; IMDC, International Metastatic RCC Database Consortium; Ipi, ipilimumab; ITT, intention to treat; Lenva, lenvatinib; NA. not available; Nivo, nivolumab; NR, not reached; ORR, objective response rate; OS, overall survival; PD, progressive disease; PD-L1, programmed cell death ligand-1; Pem, pembrolizumab; PFS, progression-free survival; PR, partial response; SD, stable disease; Sun, sunitinib; TRAE, treatment related adverse event; VEGFi, vascular endothelial growth factor inhibitor.

**Table 2:** Summary of PFS and OS of front-line pivotal trials according to PD-L1 expression.

	CheckMate 214 (Int/Poor) [16,18]	IMmotion151 [30,31]	KEYNOTE-426 [25,26]	JAVELIN Renal 101 [28,29,38]	CheckMate 9ER [32]	CLEAR [34]
	Ipi+Nivo vs. Sun	Atezo+Bev vs. Sun	Pem+Axi vs. Sun	Avel+Axi vs. Sun	Nivo+Cabo vs. Sun	Pem+Lenva vs. Sun
PD-L1 positive rates (%)	26% vs. 29%	39% vs. 40%	59% vs. 62%	61% vs 65%	26% vs. 25%	30% vs. 33%
PD-L1 cut off	1%, TC	1%, IC	1%, tumor CPS	1%, IC	1%, TC	1%, tumor CPS
PD-L1 antibody	Dako PD-L1 28-8 pharmDx.	VENTANA PD-L1 SP142 assay	PD-L1 22C3 pharmDx assay	PD-L1 SP263 assay	Dako PD-L1 28-8 pharmDx.	PD-L1 22C3 pharmDx assay
OS in PD-L1 positive (HR, 95% CI)	0.45 (0.29–0.71)	0.68 (0.46–1.00)	0.54 (0.35–0.84)	0.83 (0.60–1.15)	0.80 (0.48–1.34)	0.76 (0.46–1.27)
OS in PD-L1 negative (HR, 95% CI)	0.73 (0.56–0.96)	NA	0.59 (0.34–1.03)	NA	0.51 (0.34–0.75)	0.50 (0.28–0.89)
OS in ITT (HR, 95% CI)	0.63 (0.44–0.89)	0.81 (0.63–1.03)	0.53 (0.38–0.74)	0.80 (0.62–1.03)	0.60 (0.40–0.89)	0.66 (0.49–0.88)
PFS in PD-L1 positive (HR, 95% CI)	0.46 (0.31–0.67)	0.74 (0.57–0.96)	0.62 (0.47–0.80)	0.62 (0.49–0.78)	0.49 (0.32–0.73)	0.40 (0.27–0.58)
PFS in PD-L1 negative (HR, 95% CI)	1.00 (0.80–1.26)	NA	0.87 (0.62–1.23)	NA	0.52 (0.40–0.67)	0.39 (0.26–0.59)
PFS in ITT (HR, 95% CI)	0.82 (0.64–1.05)	0.83 (0.70–0.97)	0.69 (0.57–0.84)	0.69 (0.57–0.83)	0.51 (0.41–0.64)	0.39 (0.32–0.49)

Atezo, atezolizumab; Avel, avelumab; Axi, axitinib; Bev, bevacizumab; Cabo, cabozantinib; CI, confidence interval; Ever, everolimus; HR, hazard ratio; IC, immune cell; Ipi, ipilimumab; ITT, intention to treat; Lenva, lenvatinib; NA. not available; Nivo, nivolumab; OS, overall survival; PD-L1, programmed cell death ligand-1; Pem, pembrolizumab; PFS, progression-free survival; Sun, sunitinib; TC, tumor cell.

**Table 3:** Summary of efficacy of front-line ICI-based treatment in patients with sarcomatoid feature.

	CheckMate 214 (Intermediate/Poor) [46]		IMmotion151 [51]		KEYNOTE-426 [47]		CheckMate 9ER [48]		CLEAR [49]	
	Ipi+Nivo (*n* = 74)	Sun (*n* = 65)	Atezo+Bev (*n* = 68)	Sun (*n* = 74)	Pem+Axi (*n* = 46)	Sun (*n* = 50)	Nivo+Cabo (*n* = 34)	Sun (*n* = 41)	Pem+Lenva (*n* = 28)	Sun (*n* = 21)
ORR (%)	61	23	49	14	58.8	31.5	54.8	28.4	60.7	23.8
CR (%)	19	3	10	3	13	2	9.3	4.3	NA	NA
PFS (months)	26.5	5.1	8.3	5.3	NR	8.4	10.3	4.2	11.1	5.5
HR (95% CI)	0.54 (0.3–0.9)		0.52 (0.34–0.79)		0.54 (0.29–1.00)		0.42 (0.23–0.74)		0.39 (0.18–0.84)	
OS (months)	NR	14.2	21.7	15.4	NR	NR	NR	19.7	NR	NR
HR (95% CI)	0.45 (0.3–0.7)		0.64 (0.41–1.01)		0.58 (0.21–1.59)		0.36 (0.17–0.79)		0.91 (0.32–2.58)	

Atezo, atezolizumab; Avel, avelumab; Axi, axitinib; Bev, bevacizumab; Cabo, cabozantinib; CI, confidence interval; CR; complete response; HR, hazard ratio; ICI, immune checkpoint inhibitor; Ipi, ipilimumab; Lenva, lenvatinib; NA, not available; NR, not reached; Nivo, nivolumab; OS, overall survival; ORR, objective response rate; Pem, pem-brolizumab; PFS, progression-free survival; Sun, sunitinib.

**Table 4:** Summary of selected potential candidates for novel treatment of advanced ccRCC.

Agent	Phase	Target/Mechanism	NCT Number
MK2206	1	AKT Inhibitor	NCT01480154
DS-6000a	1	Antibody Drug Conjugate (CDH6)	NCT04707248
Ibrutinib	1/2	BTK inhibitor	NCT02899078
APL-501	1/2	C-MET inhibitor	NCT03655613
CCT301	1/2	CAR-T	NCT03393936
ALLO-316	1	CAR-T	NCT04696731
CAIX-targeted CAR-T Cells	1	CAR-T	NCT04969354
Abemaciclib	1	CDK4/6 inhibitor	NCT03905889, NCT04627064
Palbociclib	2	CDK4/6 inhibitor	NCT05176288
CMN-001	2	Cell therapy (Dendritic cell)	NCT04203901
CTX130	1	Cell therapy (T cell)	NCT04438083
HERV-E TCR transduced autologous T cells	1	Cell therapy (T cell)	NCT03354390
PF-04518600	2	Checkpoint (Anti-OX40 antibody)	NCT03092856
Daraturumab	1	Checkpoint (Anti-CD38 monoclonal antibody	NCT03473730
APX005M	1	Checkpoint (CD40 agonistic monoclonal antibody)	NCT04495257, NCT03502330
MEDI5752	1	Checkpoint (Monovalent Bispecific PD-1/CTLA4 Antibody)	NCT04522323
Cyclophosphamide	1/2	Chemotherapy (metronomic cyclophosphamide)	NCT04262427
X4P-001	1/2	CXCR4 antagonist	NCT02667886
DS3201	1,2	EZH1/2 Dual Inhibitor	NCT04388852
Entinostat	1	HDAC inhibitor	NCT03552380, NCT03501381, NCT01038778
HBI-8000	1/2	HDAC inhibitor	NCT02718066
Vorinostat	1	HDAC inhibitor	NCT02619253
Seleno-L-methionine	1/2	HIF1/2 degradation enhancer	NCT02535533
ARO-HIF2	1	HIF2 RNA interference molecule	NCT04169711
Belzutifan	1,2	HIF2a inhibitor	NCT03634540, NCT04489771, NCT03401788, NCT04195750, NCT04846920
NKT2152	1/2	HIF2a inhibitor	NCT05119335
PT2385	2	HIF2a inhibitor	NCT03108066
Epacadostat	3	IDO1 inhibitor	NCT03260894
TAK-228	2	mTOR1/2 inhibitor	NCT03097328
Anlotinib	2	Multi-target TKI	NCT02072044
Sitravatinib	1	Multi-target TKI	NCT04518046
ESK981	2	Multi-target TKI	NCT03562507
Sitravatinib	2	Multi-target TKI	NCT04904302
NeoVax	1	Neoantigen long peptide vaccines	NCT02950766
Talazoparib	2	PARP inhibitor	NCT04068831, NCT04337970
Olaparib	2	PARP inhibitor	NCT03786796, NCT03741426
IPI-549	2	PI3K-γ inhibitor	NCT03961698

AKT, phosphatidyl-inositol-3-kinase−protein kinase B; BTK, Bruton’s tyrosine kinase; CAR-T, chimeric antigen receptor-T; CDH6, cadherin-6; ccRCC, clear cell renal cell carcinoma; CDK, cyclin-dependent kinase; C-MET, hepatocyte growth factor receptor; CTLA4, cytotoxic T lymphocyte antigen-4; CXCR4, C-X-C motif chemokine receptor 4; EZH, enhancer of zeste homologue; HDAC, histone deacetylase; HERV-E, human endogenous retrovirus-E; HIF, hypoxia inducible factor; IDO1, indoleamine 2,3-Dioxygenase 1; mTOR, mammalian target of rapamycin; OX40, CD134; PARP, Poly (ADP-ribose) polymerase; PD-1, programmed cell death-1; PI3K-γ, phosphoinositide 3-kinase-gamma; TCR, T cell receptor; TKI, tyrosine kinase inhibitor.
